# What Are They Up To? The Role of Sensory Evidence and Prior Knowledge in Action Understanding

**DOI:** 10.1371/journal.pone.0017133

**Published:** 2011-02-18

**Authors:** Valerian Chambon, Philippe Domenech, Elisabeth Pacherie, Etienne Koechlin, Pierre Baraduc, Chlöé Farrer

**Affiliations:** 1 Centre de Neuroscience Cognitive, Université de Lyon, CNRS, Bron, France; 2 Institut Jean Nicod, EHESS, DEC-ENS, CNRS, Paris, France; 3 INSERM, Ecole Normale Supérieure, Université Pierre et Marie Curie, Paris, France; 4 Centre de Recherche Cerveau et Cognition, Université de Toulouse, UPS-CNRS, Toulouse, France; 5 Faculté de Médecine de Rangueil, Toulouse, France; French National Centre for Scientific Research, France

## Abstract

Explaining or predicting the behaviour of our conspecifics requires the ability to infer the intentions that motivate it. Such inferences are assumed to rely on two types of information: (1) the sensory information conveyed by movement kinematics and (2) the observer's prior expectations – acquired from past experience or derived from prior knowledge. However, the respective contribution of these two sources of information is still controversial. This controversy stems in part from the fact that “intention” is an umbrella term that may embrace various sub-types each being assigned different scopes and targets. We hypothesized that variations in the scope and target of intentions may account for variations in the contribution of visual kinematics and prior knowledge to the intention inference process. To test this hypothesis, we conducted four behavioural experiments in which participants were instructed to identify different types of intention: basic intentions (i.e. simple goal of a motor act), superordinate intentions (i.e. general goal of a sequence of motor acts), or social intentions (i.e. intentions accomplished in a context of reciprocal interaction). For each of the above-mentioned intentions, we varied (1) the amount of visual information available from the action scene and (2) participant's prior expectations concerning the intention that was more likely to be accomplished. First, we showed that intentional judgments depend on a consistent interaction between visual information and participant's prior expectations. Moreover, we demonstrated that this interaction varied according to the type of intention to be inferred, with participant's priors rather than perceptual evidence exerting a greater effect on the inference of social and superordinate intentions. The results are discussed by appealing to the specific properties of each type of intention considered and further interpreted in the light of a hierarchical model of action representation.

## Introduction

### Intentional inference: perceptual information and top-down prior knowledge

Explaining or predicting the behaviour of our conspecifics requires the ability to properly appreciate the causes that motivate it. As these causes are hidden – intentions, like beliefs or desires, are unobservable states –, it has long been a matter of speculation how one may infer them from patterns of visible behaviour alone. Indeed, visual information conveyed by the movement kinematics is often noisy, ambiguous or incomplete. As a result, visual information generally under-constraints the space of candidate causes (i.e. the many competing intentions) that are logically consistent with what is observed [1]–[3]. One way to solve this problem is to assume that this space of possible intentions is further constrained by the observer's prior expectations. These expectations are derived from prior knowledge that may originate from the past experience of the viewer (through expertise: [4], [5]; or learning of statistical regularities: [6]), from her intuitive theories [7], [8], or reputational knowledge [9], [10], as well as from contextual information surrounding the action scene [5], [11]. This prior knowledge has been demonstrated to be crucial to account for the robustness of our everyday inferences [12]. Indeed, it makes possible inductive inference about the agent's intentions, even in cases of noisy signals or incomplete data [13]–[15].

However, although most authors agree that prior knowledge and perceptual information both contribute to the process of inferring intentions, the precise contribution of each type of information remains controversial [16]–[23]. The controversy stems in part from the fact that “intention” is an umbrella term used in the empirical and philosophical literature to refer to representations of actions that can differ in both their content and format, as well as in their temporal properties and in the role they play in the guidance of actions [24]–[29]. Intentions can therefore be distinguished into several sub-types according to one or several of these factors. In the present study, we propose a typology of intentions and hypothesize that this typology might be a key element in understanding how perceptual information and prior knowledge contribute to the process by which intentions are inferred.

### Varieties of intentions

The intentional typology we present below is primarily motivated by the necessity to take into account two dimensions of variation in the content of intentions that may make an important difference to the processes involved in their inference. The first dimension of variation concerns the scope of the intention; i.e., the more-or-less complex nature of its goal. Here we can draw a distinction between *basic intentions* and *superordinate intentions*. *Basic intentions* are directed at simple motor goals (i.e. goals that can be realized by basic actions such as lifting an arm, pressing a button, or reaching for an object). These intentions are sensorimotor representations where the goal is represented directly in terms of the motor commands needed to achieve it. The relation between basic intention and motor act is thus one-to-one when that act is successfully completed [17]. In contrast, s*uperordinate intentions* are intentions directed at somewhat more complex or general goals, the achievement of which typically involves the completion of a number of subgoals or substeps. Depending on the complexity of the general goal, these subgoals may themselves be decomposed into further subgoals, ultimately reaching the level of basic actions. The achievement of a superordinate intention will thus require the execution of a combination of basic actions each guided by a corresponding basic intention. Different combinations of motor acts can be used to accomplish the same general goal and, conversely, a same motor act (or even series of motor acts) can be part of combinations aimed at different general goals.

The second dimension of variation we were interested in concerns the target of the intentions. Neither basic nor superordinate intentions are necessarily directed at inanimate objects. They may also target a third party or be achieved in a context of social interaction [11], [30]–[33]. The content of intentions is thus also modulated by the relational structure in which an action takes place. We call intentions directed at an object, *non-social* intentions, and intentions directed at a third party, *social* intentions.

By combining these two dimensions, we obtain the following typology: i) *non-social* basic intention, ii) *non-social* superordinate intention, iii) *social* basic intention, and iv) *social* superordinate intention. Owing to their different scopes and targets, basic and superordinate, social and non-social, intentions are naturally assigned different functional roles, different types of content and different temporal scales. The present study aims at investigating whether these functional differences are reflected in the respective contribution of perceptual information and prior expectations to intentional judgments.

### Overview of the present study

We conducted four experiments in which participants were requested to identify one intention underlying an action scene. Each experiment involved one type of intention with a specific scope (basic *vs.* superordinate) and a specific target (social *vs.* non-social). Interactions between prior expectations and visual information were examined within a Bayesian probabilistic framework. This conceptual framework is particularly well-suited to account for how accurate predictions on hidden world states are made in situations where available sensory information does not sufficiently constrain the number of potential solutions [13], [15], [34]. Before the onset of an action sequence, each of the agent's possible intentions was first assigned a certain ‘level of belief’, termed *a priori probability* (the probability that intention X is the real cause of the observed behaviour estimated from past experiences). Then the observer progressively gathers sensory information (visual input) as the action sequence is disclosed and both sources of information (sensory and *a priori*) are combined and used to infer the intention underlying the observed behaviour. Thus, the process by which intentions are inferred is considered as reflecting a trade-off between the sensory information and the prior probability of each candidate intention [14]. Finally, the chosen intention is that which maximizes the *posterior probability* value, *i.e.* the probability that intention X is true *given* what is observed.

In the present study, these two terms – *a priori* probability and sensory information – were manipulated using a two-step procedure. Prior expectations the participants had about the agent's possible intentions were manipulated by increasing the *a priori* probability that one intention (termed *likely intention*) was achieved, to the detriment of other intentions (*unlikely intention*s) with the same scope and target. Sensory information available from an action scene was then manipulated in a second step by modulating the degree of completeness (i.e. the duration) of the action sequences, resulting in actions scenes with varying amounts of visual information.

We first predicted that judgements about intentions would follow the general principles of Bayesian inference. Specifically, we expected that the amount of visual information would interact with participants' prior expectations such that the lower the reliability of the external visual input, the more participants' responses would depend upon their own internal expectations. That is, they should respond more frequently in the direction of the *likely* intention. And vice versa, the higher the amount of visual information, the less the participants should rely on their prior expectations.

Second, we predicted that the shape of the interaction between these two sources of information would be a function of the *type* of intention, depending on both its scope and target. Along the dimension of the scope, we hypothesised that participants' judgement about basic intentions should primarily rely on sensory information available from the action scene. This prediction is motivated by the pragmatic content of the basic intention: “grasping a glass of water” directly denotes the corresponding intention of “grasping that glass”. In this case, perceiving the action itself – i.e. processing the associated visual kinematics – is enough to successfully determine the nature of the underlying intention [35]. On the other hand, we expected performance in judging superordinate intentions to be significantly influenced by participants' prior expectations. As already mentioned, the same sequence of motor acts can be part of combinations aimed at different general goals or superordinate intentions. In this specific case, sensory information carried by movement kinematics is not sufficient to infer the corresponding intention, as it under-constraints the set of candidate intentions congruent with this movement [1], [2], [17], [36]. We consequently predicted that this perceptual uncertainty should encourage participants to ‘mistrust’ what they observe and, hence, to rely more on their prior expectations.

Along the dimension of target, finally, we expected participants' reliance on their prior expectations to increase when basic and superordinate intentions are directed at another agent. The structure of social interaction meets indeed particular, often irrepressible, expectations, such as those provided by reputational knowledge [9], [10], [37]. Indeed, knowledge about individual's reputation has been robustly demonstrated to have a strong impact on predicting how the observed agent will behave [12]. In line with other recent suggestions, we thus hypothesised that the weight of these *a priori* expectations would increase when the observed action fits into a context of social interaction.

## Materials and Methods

### Non-social experiments

**Figure 1:**
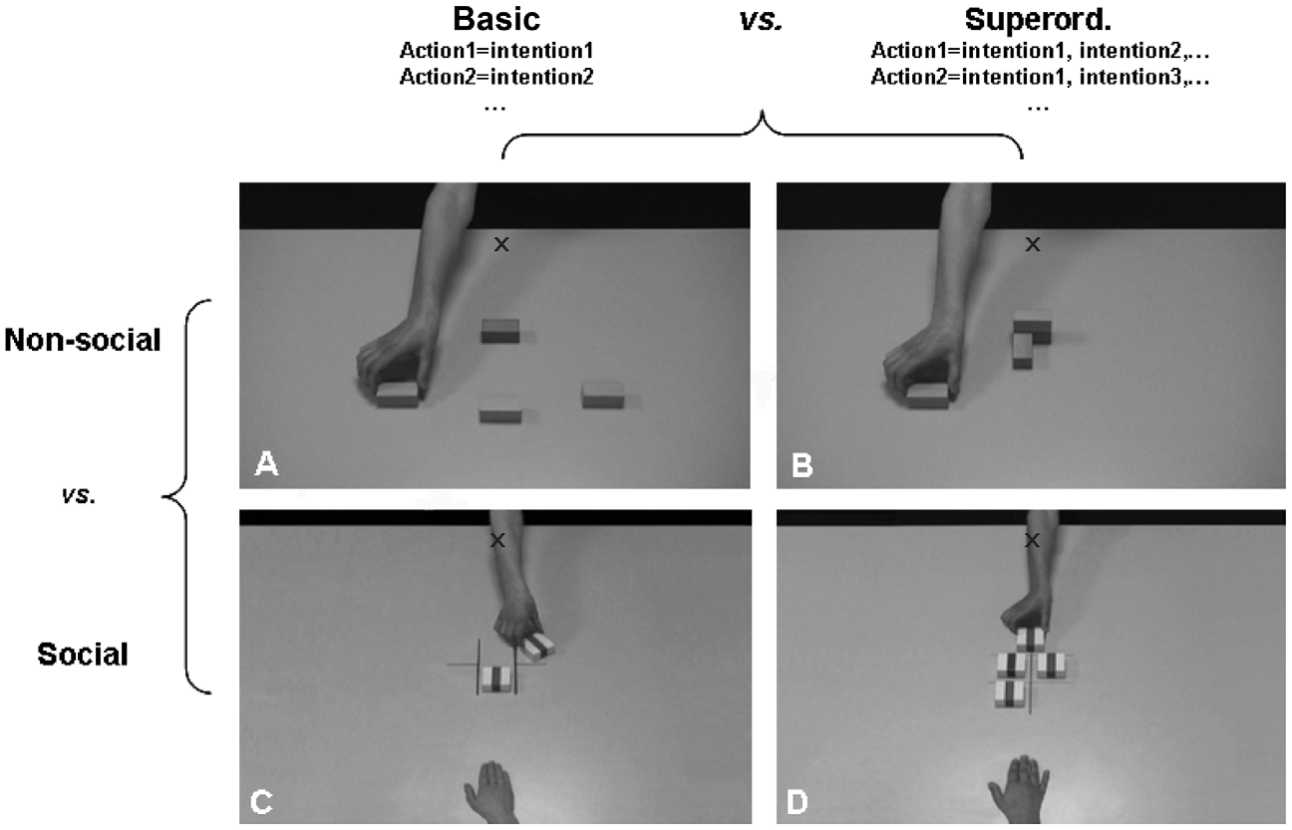
Examples of stimuli for each of the four experiments. basic non-social intention experiment (**A**); superordinate non-social intention experiment (**B**); basic social intention experiment (**C**); superordinate social intention experiment (**D**). The black cross indicates the starting position of the hand.

In the first experiment, participants were instructed to infer the *basic intention* (to lift, to rotate, or to transport) of an actor manipulating meaningless objects (fig. 1, **A**). In the second experiment, participants were instructed to infer the *superordinate intention* (i.e. the general goal) underlying a sequence of motor acts (fig. 1, **B**).

### Social experiments

The third (fig. 1, **C**) and fourth experiments (fig. 1, **D**) presented two actors engaged in a social game in which they could either cooperate or defect by coordinating their action (joint-action condition) or by refusing to join their action to the achievement of a shared goal (defective condition). Participants were instructed to infer the nature of the second player's social intention (i.e. cooperative or defective intention). In both these experiments, the bias was assigned according to the way the second player responded to the strategy adopted by the opponent in the previous round. Participants were therefore biased towards the reputation of the second player rather than towards one particular type of social intention. Finally, as in the two previous non-social experiments, both *basic intentions* (single motor acts) and *superordinate intentions* (sequences of motor acts leading to the construction of a shape) were considered in the last two experiments.

### Ethics Statement

All participants gave written informed consent for the study which was approved by the local Ethical Committee (Comité de Protection des Personnes SUD-EST IV, no. B80631-60).

### Experiment 1: non-social basic intention

#### Participants

30 healthy subjects (15 males, 15 females, mean age = 35.13, S.D. = 9.33 and laterality score mean = 0.88, S.D. = 0.31; [38]) participated in this experiment. They all reported normal or corrected-to-normal visual acuity. Participants received 10 euros for taking part in the study.

#### Stimuli

Visual stimuli were incomplete movies representing an actor's hand performing a simple manipulation of a meaningless object. The duration of the video sequences was varied on 4 distinct levels, ranging from 1480 ms to 1880 ms after movement onset. Each movie was characterized by one basic intention (to transport, to rotate, or to lift an object) and participants were instructed to infer the basic intention in each video. There were three white rectangular objects of similar size (3 cm×6 cm) and orientation, positioned at equal distance (16.8 cm) from the starting position of the actor's hand (figure 1, A). All the movies were performed by a single actor and only featured her naked arm. Each action was performed as often with one object as with the others.

Movies were equalized for temporal homogeneity (see **Supporting Text S1, part 1,** and **Figure S1**). Furthermore, all the movies were unique, i.e., they were presented only once to prevent any influence of memorized kinematic parameters on participants' performance in the experiment.

#### Procedure

Participants were comfortably sat at a distance of 60 cm from a 19″ computer monitor. Each trial started with the observation of an incomplete movie, and then a response screen appeared for 2500 ms representing the first letter of each possible intention (T for ‘transport’, L for ‘lift’, or R for ‘rotate’). Participants were requested to respond by pressing, as quickly and accurately as possible, one of the three keyboard presses corresponding to the three possible intentions. Once a response was given, the next trial started.

The design was composed of two experimental sessions. A first baseline session was characterized by a flat (unbiased) prior probability distribution with all basic intentions having the same probability. In a second bias session, participants were biased towards one intention (likely intention, 55%) to the detriment of the others (unlikely intentions, 22% each). The bias was randomly assigned so that each basic intention was equally biased across participants.

**Figure 2:**
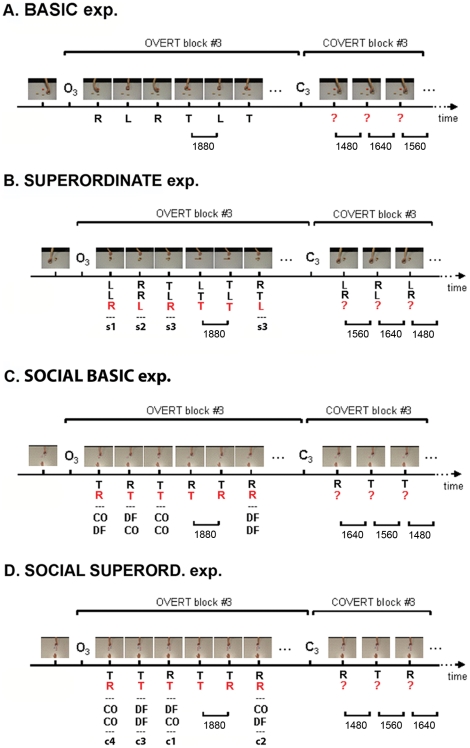
Experimental designs. Examples of a typical experimental sequence (one OVERT block followed by one COVERT block) used in both the baseline and the bias sessions. *OVERT blocks (O)*: 18 movies with a very high and constant amount of visual information (1880 ms). *COVERT blocks (C)*: 9 movies with three amounts of visual information (1480, 1560, and 1640 ms). In the 4 experiments, the probability of all intentions was held equal across the block, except in overt blocks of the bias session, where one particular intention had a greater probability to be accomplished than the other ones. **In the BASIC exp.**, subjects had to identify a single action (*labels*: **L**: “lift” action; **R**: “rotate” action; **T**: “transport” action). **In the SUPERORDINATE exp.**, subjects had to identify the final action (red letter) of a sequence leading to *shape 1, 2,* or *3* (**s1**: shape 1). **In the SUPERORD. SOCIAL exp.**, subjects had to identify the action of the second player (red letter) leading to *configuration 1, 2*, *3,* or *4* (**c1**: configuration 1). In both **BASIC and SUPERORD. SOCIAL exp.**, the action or the configuration achieved by each player indicated either a cooperative or a defective strategy (**CO**: cooperate; **DF**: defect). In each experiment, a probabilistic bias was assigned to one particular action (basic), shape (superordinate) or strategy (social). The red interrogation mark indicates the action (basic: single action; superordinate: last action of the sequence) for which the amount of visual information varied.

In both sessions, trials were organized into OVERT blocks in which movies consisted of a constant and very high amount of visual information (1880 ms), alternating with COVERT blocks in which movies consisted of varying and lower amounts of visual information presented in a random order (low = 1480 ms, moderate = 1560 ms, and high = 1640) (see **Supporting Text S1, part 2**, for the selection of these amounts of visual information[58], [59]; **Figure S2**). Each experimental sequence (one overt block followed by one covert block) was repeated 9 times over each session (see figure 2, A) and each participant performed the trials in a different random order.

The reason for block interleaving was that it enabled us to maintain the bias constant across the bias session. Indeed, by regularly inserting overt blocks of movies with different probabilities, we ensured that participants were continuously biased towards one intention over the whole session. Furthermore, even though the baseline session did not include any bias assignment, and therefore was not concerned with bias maintenance, it contained the same trial organization (block interleaving) to allow a direct comparison of the performance between the two sessions.

Prior to running the experiment, participants undertook a training session to get familiar with the task. The training consisted of 3 baseline experimental sequences (non-biased probability distribution) with interleaved blocks. The 72 movies (3×24) presented during the training session were distinct from those used in the experiment.

#### Design and statistical analyses

One group of statistical analyses was performed for each session independently (baseline and bias sessions), on the two dependant variables (participants' hits and reaction times for correct responses). In the overt blocks of the baseline session, one two-tailed t-test was conducted on participants' reaction times (RTs) between the future ‘likely’ intention (the one towards which participants were subsequently biased in the bias session) and the future ‘unlikely’ intentions. The same test was conducted in the bias session between likely and unlikely intentions. In the covert blocks, a 2×3 repeated-measures ANOVA was performed for each session on both RTs and hits. The first two-level factor was the bias (future ‘likely’ vs. future ‘unlikely’ intentions OR likely vs. unlikely intention) and the second three-level factor was the amount of visual information (low, moderate and high). Post-hoc Fisher tests were then performed to identify differences between conditions.

Another group of analyses was conducted in order to assess the magnitude of the bias effect on participants' performance. To do so, we looked at whether increasing the probability of one intention concomitantly affected the selection of intentions with lower probability. Two-tailed t-tests on RTs and hits for unlikely intentions were thus performed between the baseline and the bias sessions. We predicted that selecting an unlikely intention should be more demanding in the bias session – as it concomitantly requires inhibiting a competing biased intention – than in the baseline session, where all intentions had the same probability of occurring. In the following, the resulting “cost” (i.e. increased RTs and decreased hits for unlikely intentions) was considered as an indirect measure of the bias effect.

For all analyses, a p<.05 was taken as the criterion for significance and an eta squared (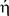) was used as a measure of effect size. These analyses were performed using the statistical software *Statistica 7* (www.statsoft.com).

### Experiment 2: non-social superordinate intention

#### Participants

30 new participants (15 males, 15 females, mean age = 36.59, S.D. = 8.12 and laterality score mean  = 0.79, S.D. = 0.19) took part in this experiment. They all reported normal or corrected-to-normal visual acuity and received 10 euros for taking part in the study.

#### Stimuli

As with the non-social basic experiment, test material consisted of incomplete movie clips showing an actor's hand manipulating meaningless objects. However, contrary to experiment 1, movies in the superordinate experiment represented a sequence of *three* successive manipulations (to transport, rotate, or lift the object) leading to the construction of a meaningless shape. Each sequence was therefore characterized by an underlying superordinate intention, represented by one final shape (s1, s2 or s3). The objects used in the first experiment were also used in this second experiment (figure 1, B). The first action was performed on one of the three objects, the second action on one of the two objects left, and the third action on the remaining object. After each action, the hand came back to the starting position. The incompleteness of the video sequences was controlled so that the duration of the last action was varied on 3 distinct levels (1480 ms, 1560 ms or 1640 ms after this action starts). All the movies were made with the same actor as in experiment 1. Finally, temporal homogeneity of the movies was controlled and each trial was unique.

#### Procedure

The organization of the trials was the same as in the non-social basic experiment (see figure 2, B, ‘Superordinate exp.’). However, the task was further constrained. First, to ensure that participants paid attention to the overall sequence of actions, they were asked to identify what the last (not-yet completed) action of this sequence was, by pressing as quickly and accurately as possible, one of the three corresponding keyboard presses (T, L, or R). This response depended upon having inferred the superordinate intention of this sequence (i.e. the final shape being achieved by a set of three successive actions). Second, to ensure that participants were biased towards the superordinate intention itself and not towards the last final action only, commutative sequences were used so that each shape could be constructed from distinct sequences of actions sets. Sequences shown in the covert blocks were thus distinct from those used in the overt blocks (e.g. the shape s1 could be obtained from the sequence ‘lift-lift-rotate’ in an overt block, but from the sequence ‘lift-rotate-lift’ in a covert block). Finally, the intention could not be inferred solely from the motor acts composing the sequence. Indeed, although the probability of each shape being constructed was manipulated in the bias session (i.e. one particular shape had a higher probability), the probabilities of the individual actions (lift, rotate, or transport) were held equal at each step of that sequence. Finally a training session was conducted with movies distinct from those used in the experimental sessions.

### Experiment 3: social basic intention

#### Participants

30 novel subjects (15 males, 15 females, mean age  = 32.9, S.D. = 10, and laterality score mean  = 0.80, S.D. = 0.11) participated in this experiment. They all reported normal or corrected-to-normal visual acuity and received 10 euros for taking part in the study.

#### Stimuli

Visual stimuli consisted of incomplete movies showing two players' hands (opposite each other) manipulating meaningless objects. Two objects (printed with a blue or a red line) were placed on the sides of a grid that was situated in the centre of the scene (figure 1, C). The objects were of similar size (5.8 cm×5.8 cm) and located at equal distance from the starting position of each player's hand (figure 1, C). The two actors played one after the other by moving the object towards the middle of the grid (termed ‘bank’) or by rotating it so that it remained at its own place. Movies were partitioned so that the last action (i.e. the action performed by the second player) was incomplete (1480 ms, 1560 ms, or 1640 ms after the last action starts).Here, each motor act directly denoted the social intention of the player: each player could either cooperate with the other one, by moving the object towards the central bank (transport), or defect, by leaving the object at its own place (rotate). Consequently, there were four possible combinations of intentions, or strategies: either both players cooperated (transport/transport) or defected (rotate/rotate), or the first player defected and the second cooperated (rotate/transport), or the first player cooperated and the second defected (transport/rotate). Finally, temporal homogeneity of the movies was controlled and each trial was unique.

#### Procedure

For each trial, participants were instructed to observe the incomplete movie and infer what the last action (i.e. the one performed by the second player) was. This response required the participant to have inferred the second player's intention (to defect or to cooperate) which itself depended upon the first player's strategy. Participants were asked to give their answer by pressing, as quickly and accurately as possible, one of the two keyboard keys corresponding to the two possible last actions (T for ‘transport’, or R for ‘rotate’) susceptible to achieve the second player's intention. Once a response was given, the next trial started.

In the baseline session, all combinations of strategies were counterbalanced over the blocks (i.e. whatever the first player did the second player was just as likely to defect or to cooperate). In the bias session, on the other hand, the probability that the second player did whatever the opponent did in the previous round was increased, thus biasing participants to perceive the second actor as a “tit-for-tat” player (i.e. as being more inclined to cooperate if the first player had previously cooperated, and to defect if the first player had previously defected). The rationale for biasing the second player's reputation in such a way was twofold. First, tit-for-tat (TFT) reputation implies that individuals respond to their opponent's actions in a mirrored (i.e. correlated) fashion. Therefore, successfully predicting intentions of a TFT-like player necessarily involves taking into account what the first player has done, and by consequence, ensured that participants paid attention to the whole sequence of actions (both actor 1′s play and actor 2′s play). Second, contrary to other common types of reputation such as “always defect”, or “always cooperate”, TFT may equally imply cooperative as well as defective strategies. The probability that the second actor behaves as a tit-for-tat player could thus be increased without otherwise increasing the probability of one intention (e.g. cooperate) to the detriment of the other one (e.g. defect). Holding equal the probability of both these strategies was here crucial to nullify their potential kinematic differences on participants' performance (see [39]) and also to avoid stereotyped responses (e.g. always responding ‘cooperate’ or ‘defect’). Finally, a tit-for-tat strategy is known to be a more intuitive and successful strategy than alternative ones, such as “always cooperating”, “always defecting” or “acting randomly” [40]–[42]. We thus chose to experimentally strengthen this already existing *a priori* bias by increasing the probability that the second player's action mirrors her opponent's one while holding equal both the probability of each single act (to rotate or to transport) and the overall probability of each intention (to defect or to cooperate) (figure 2, C, ‘Social Basic exp.’). Thus, in the baseline session, the second player was as likely to play tit-for-tat as she was to play the other types of strategy. In the bias session, however, the probability that the second actor played tit-for-tat was increased so that she was more likely to cooperate (rather than defect) if the first player had previously cooperated, and to defect (rather than cooperate) if the first player had previously cooperated. Finally a training session was conducted with movies distinct from those used in the experimental sessions.

### Experiment 4: social superordinate intention

#### Participants

30 novel participants (15 males, 15 females, mean age  = 34.27, S.D. = 9.42, and laterality score mean  = 0.83, S.D. = 0.26) participated in this last experiment. They all reported normal or corrected-to-normal visual acuity and received 10 euros for taking part in the study.

#### Stimuli

As in the social basic experiment, the stimuli of this last experiment represented two players' hands manipulating objects. However, in the present experiment, the actors played in turn with the goal of vertically aligning three objects (see figure 1, D). The goal of the first player was to align the objects according to the color (red), irrespective of the orientation, while the goal of the second player was to align the objects according to the orientation, irrespective of the color. A third configuration could be obtained by the alignment of the objects according to both the orientation and the color. As in the social basic experiment, the two social intentions were of a defective or a cooperative nature. However, in the present experiment, the social intention was denoted by the sequence of the players' motor acts (i.e. the final configuration), rather than by the single action performed by each player. Indeed, a defective or a cooperative strategy could be equally achieved by rotating or transporting the object. Each player could adopt a defective strategy by manipulating the object in such a way that it prevented the creation of one configuration, or adopt a cooperative strategy in order to achieve another configuration. The four possible final configurations were therefore: either both players defected (configuration 3) or cooperated (configuration 4), or the first player defected and the second cooperated (configuration 1), or the first player cooperated and the second defected (configuration 2). As for previous experiments, only the last action (i.e. the action performed by the second player) was made incomplete (1480 ms, 1560 ms or 1640 ms). Finally, temporal homogeneity of the movies was controlled and each trial was unique.

#### Procedure

Participants were instructed to infer the social superordinate intention of the second player by indicating which action allowed the accomplishment of that intention. A correct response therefore required having correctly inferred the second player's intention (to defect or to cooperate) which itself depended upon the first player's strategy. Participants were asked to give their answer by pressing, as quickly and accurately as possible, one of the two keyboard keys corresponding to the two possible actions (T for ‘transport’ or R for ‘rotate’) congruent with the second player's social intention. The organization of trials in the social superordinate experiment was the same as for the social basic experiment (see figure 2, D). Tit-for-tat reputation (i.e. the second player did whatever the opponent did in the previous round) was chosen to be biased across participants. Likewise, commutative sequences were also used so that each pattern could be obtained from distinct sequences of actions ensuring that the different strategies could not be predicted from motor acts solely. Furthermore, both the overall probability of each strategy (cooperative or defective) and each action (to rotate or to transport) were held equal across the blocks. Finally a training session was conducted with movies distinct from those used in the experimental sessions.

### ‘Bias effect’: between-experiment comparisons

Finally, some analyses were conducted to directly assess whether the contribution of prior knowledge to the inference of an intention depended upon the target (basic vs. superordinate) and the scope (non-social vs. social) of the intention. Student tests were first conducted on the overall performance in the bias session between the basic experiments and the superordinate ones, and between the non-social experiments and the social ones. Second, the bias effects for each dimension (scope and target) were compared with each other. A score reflecting the effect of each type of intention (basic, superordinate, social, non-social intention) was calculated by subtracting, in each experiment, participants' hit rate for the likely intention from the hit rate for the unlikely intentions. The resulting scores were then entered in a 2 (basic vs. superordinate) ×2 (social vs. non-social) ×3 (amounts of information) factorial ANOVA.

## Results

### Experiment 1: non-social basic intention

For each session, two-tailed t-tests were performed between the two unlikely intentions on both RTs and hits. As no significant differences appeared (all p>.05), performances for these two unlikely intentions were pooled for subsequent analyses.

#### Overt blocks

**Figure 3:**
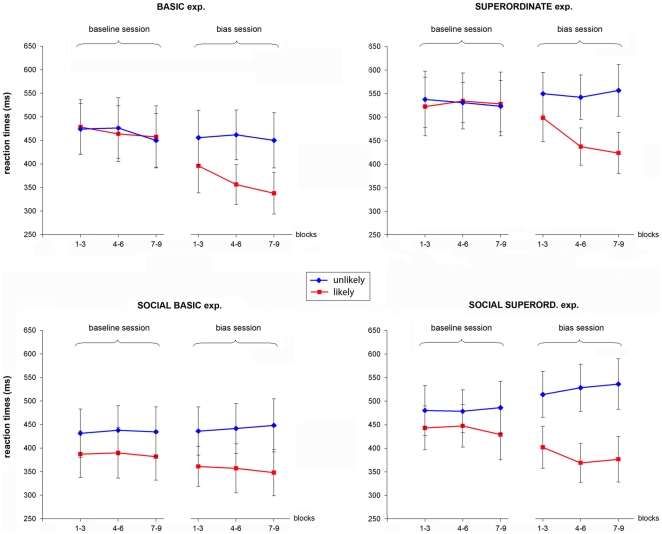
OVERT blocks: mean reaction times of the likely and the unlikely intentions across time. Baseline and bias sessions: (1-3): the first three overt blocks of the session; (4-6) the intermediate three blocks; and (7-9) the three last blocks of the session.

As expected, participants performed the task well when the amount of visual information was very high (percentage of mean correct responses  = 98%, S.D. = 2.4, and 96.8%, S.D. = 3.4 in the baseline and the bias sessions, respectively). Furthermore, in the baseline session, there were no significant differences among hits and RTs between the (future) ‘likely intention’ (i.e. the one towards which participants will be biased in the subsequent bias session) and the ‘unlikely intention’, indicating that prior to biasing participants, there was no *a priori* bias towards one intention rather than another (two-tailed *t*-tests, all p>0.2, see figure 3, ‘Basic exp.’, baseline session).

The only significant difference was found in the bias session, with faster RTs for the likely intention vs. unlikely intentions (two-tailed *t*-tests, all p<.001). Subsequent analyses of RTs across time were carried out by independently comparing RTs for likely basic intention and RTs for unlikely basic intentions across the different blocks. The bias was found to have a cumulative effect over time, with RTs for the likely intention progressively decreasing up to block 8 (minimal RT = 337 ms) and then remaining constant until the end of the session (blocks 1–3 *vs.* blocks 4–6: t = 3.09, p<.005; blocks 4–6 *vs.* blocks 7–9: t = 2.08, p<.05) (see figure 3, ‘Basic exp.’).

#### Covert Blocks

**Figure 4:**
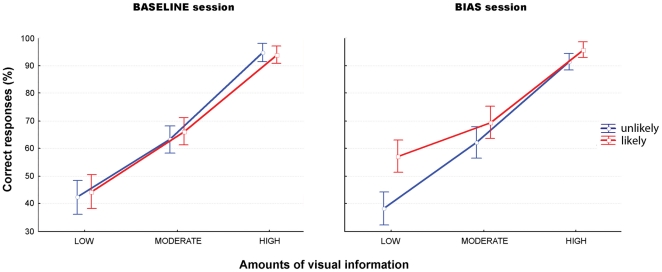
Non-social basic intention experiment (COVERT blocks). Mean percentage of correct responses (± SD) for likely (red) and unlikely (blue) intentions for each amount of visual information (low, moderate, high).

The 3 (amounts of information) ×2 (likely vs. unlikely intentions) repeated-measures ANOVA revealed significant effects on both participants' RTs and hits. In the baseline session, a significant effect of the amount of visual information was obtained (RTs: F(2,116) = 167.13, p<.001, 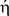 = 0.74, and hits: F(2,116) = 277.44, p<.001, 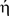 = 0.82). As expected, RTs were found to decrease and hits to improve as the amount of visual information increased. There were, however, no significant effects of the intention presented (future ‘likely’ or future ‘unlikely’ intention) nor of the interaction between the intention and the amount of visual information (both p>0.05), showing that improved performance for higher amounts of information was independent of the presented intention (Im1, Im2, or Im3) (see table 1 and figure 4, ‘Baseline session’).

**Table 1:** Non-social basic intention experiment (COVERT blocks).

Experiment		Hits (%)			RTs (ms)		
Session	Intention	LOW	MODERATE	HIGH	LOW	MODERATE	HIGH
BASIC							
Baseline	Unlikely	42.5±11.6	63.3±14.7	94.7±7	1153±303	894±205	579±193
	Likely	44.4±20.6	66±11.9	93.8±10.2	1092±288	866±247	601±143
Bias	Unlikely	38.3±14.7	62.2±15.4	91.3±8	1190±380	975±294	628±206
	Likely	57.2±17.3	69.4±15.8	95.5±7.4	817±270	659±223	552±159

Mean reaction times (*±* SD) for likely and unlikely intentions for each amount of visual information (low, moderate, high).

In the bias session, in addition to the effect of amount of visual information (RTs: F(2,116) = 98.8, p<.001, 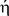 = 0.62; hits: (F(2,116) = 190.92, p<.001, 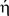 = 0.76), an effect of the bias (RTs: F(1,58) = 18.51, p<.001, 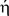 = 0.24; hits: (F(1,58) = 19.81, p<.001, 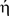 = 0.25) as well as of the interaction (RTs: F(2,116) = 13.98, p<.001, 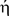 = 0.19; hits: F(2,116) = 5.44, p = .005, 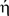 = 0.08) were also observed. Post-hoc tests indicated that participants were more accurate and faster in recognizing the likely intention in low information condition only (LSD Fisher tests, RTs: low  = p<.001; moderate  =  p<.005 and high  =  *ns*; Hits: low  = p<.001; moderate  =  *ns.* and high  =  *ns.*) (see table 1 and figure 4, ‘Bias session’).

#### Effect of the bias on the unlikely intention

We were also interested in evaluating the influence of the bias on the selection of the unlikely intentions. Comparing the performance for the unlikely intention between the two sessions revealed no significant differences for any amount of visual information (two-tailed t-tests, RTs and hits: all p>0.05). This result indicates that switching from the baseline to the bias session (i.e. increasing the probability of one intention to the detriment of others) did not significantly affect the inference of basic intentions with lower probabilities.

#### Preliminary discussion

As expected, basic intentions were better inferred as the actions were presented with a high amount of visual information. Performances were also influenced by the probability distribution of the intentions, with a significant increase in participants' hits and decrease in RTs for the likely (i.e. biased) intention. Finally, the bias significantly interacted with the amount of visual information: participants responded more often towards the likely intention when action scenes were presented with a low amount of visual information. When the amount of visual information was sufficient, prior expectations then exerted less influence on intention inference.

### Experiment 2: non-social superordinate intention

Statistical analyses were similar to those conducted in the first experiment. Responses for the two unlikely superordinate intentions were pooled for subsequent analyses as there were no significant differences among both hits and RTs between these responses (for each session, two-tailed *t*-tests, p>0.15).

#### Overt blocks

As for experiment 1, participants performed the task well when the amount of visual information was very high (1880 ms), in both the baseline (mean correct responses percentage: 98.6%, S.D. = 2.3) and the bias sessions (percentage of mean correct responses: 98.1%, S.D. = 3.1). Furthermore, in the baseline session participants were equally rapid at inferring the last action of the sequence, whatever the superordinate intention being accomplished (two-tailed *t*-tests, all p>.35).

In the bias session, however, although there were no significant differences among hits between likely and unlikely intentions, RTs for the likely intention were found to significantly decrease (two-tailed t-tests, likely *vs.* unlikely intentions, all p<.001). This decrease increased over time as revealed by a cumulative effect of the bias across blocks. Indeed, RTs decreased up to block 7 (minimal RT = 424 ms) and then remained constant until the end of the session (blocks 1–3 *vs.* blocks 4–6: t = 4.04, p<.001; blocks 4–6 *vs.* blocks 7–9: t = 0.83, p>.05) (see figure 3, ‘Superordinate exp.’).

#### Covert blocks

**Figure 5:**
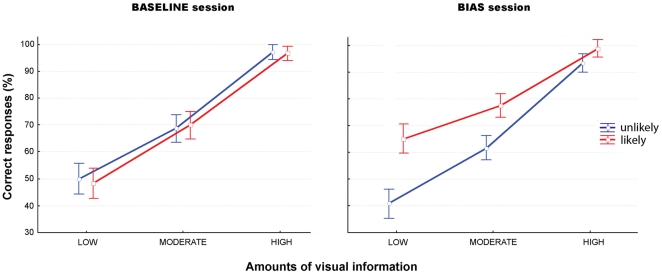
Non-social superordinate intention experiment (COVERT blocks). Mean percentage of correct responses (± SD) for likely (red) and unlikely (blue) intentions for each amount of visual information (low, moderate, high).

In both the baseline and the bias sessions, the amount of visual information significantly affected participants' hits and latencies with decreased RTs (baseline: F(2,116) = 423,68, p<.001, 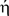 = 0.87; bias session: (F(2,116) = 523.9, p<.001; 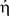 = 0.9) and a greater number of hits as the amount of visual information increased (baseline: F(2,116) = 249.18, p<.001, 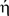 = 0.81; bias session: (F(2,116) = 199.03, p<.001; 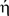 = 0.77) (see table 2 and figure 5).

**Table 2:** Non-social superordinate intention experiment (COVERT blocks).

Experiment		Hits (%)			RTs (ms)		
Session	Intention	LOW	MODERATE	HIGH	LOW	MODERATE	HIGH
SUPERORDINATE							
Baseline	Unlikely	50±15	68.7±12.1	97±6.3	1605±314	1241±218	745±153
	Likely	48.3±15.9	70±15.2	96.6±8.6	1676±304	1211±244	783±128
Bias	Unlikely	40.8±15.7	61.6±12.2	93.3±11.7	1689±307	1357±262	809±161
	Likely	65±14	77.5±12	98.7±5	1221±209	1014±183	600±138

Mean reaction times (*±* SD) for likely and unlikely intentions for each amount of visual information (low, moderate, high).

In the bias session, a significant effect of the bias was also observed with faster RTs and increased hits for actions congruent with the likely superordinate intention (RTs: F(1,58) = 47.04, p<.001, 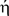 = 0.44; and hits: F(1,58) = 62.09, p<.001, 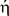 = 0.51). Finally, the bias was found to significantly interact with the amount of visual information in such a way that the number of hits was significantly higher and RTs faster for the likely intention as the amount of visual information decreased (RTs: F(2,116) = 15.3, p<.001, 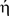 = 0.2; and hits: F(2,116) = 9.28, p<.001; 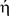 = 0.13). Post-hoc tests further indicated that participants were more accurate and faster in recognizing the likely intention in both low and moderate amount of visual information conditions (LSD Fisher: low, p<.001; moderate, p = .005, high, p<.05 for RTs; and low, p<.001; moderate, p<.001; high  =  *ns.* for hits) (see table 2 and figure 5, ‘Bias session’).

#### Effect of the bias on the unlikely intention

The number of correct responses for unlikely intentions significantly decreased in the bias session, compared to the baseline session, for both low and moderate amounts of visual information (two-tailed t-tests: all t(30)>2.33, all p<0.02). Likewise, RTs significantly decreased in the moderate amount of visual information condition (two-tailed t-tests: t(30) = −2.09, p = 0.04).

#### Preliminary discussion

Two results make the present experiment diverge from the previous one. First, the bias effect was greater in the second experiment, as it was observed in condition of low amount of visual information as well as in condition of moderate amount of information. Second, a ‘switch effect’ was also observed in both these conditions, with an increasing number of correct responses and decreasing latencies for the likely intention, accompanied by decreased hits and increased RTs for the unlikely intentions. This effect reflects the cost associated with the selection of an unlikely superordinate intention. Indeed, the increasing difficulty in disengaging from prior expectations to select a less privileged representation (i.e. unlikely intentions) reveals the greater extent to which participants rely on the bias for making their response.

### Experiment 3: social basic intention

Statistical analyses were similar to those conducted in the previous experiments. Two-tailed t-tests revealed no significant differences among both participants' RTs and hits between the TFT intentions (coop/coop *vs.* def/def: two-tailed *t*-tests, all p>.2) and between the alternative ones (coop/def *vs.* def/coop, two-tailed *t*-tests, all p>.15). Performances for def/def were therefore pooled with those of coop/coop (i.e. TFT or likely intentions) and performances for def/coop were pooled with those of coop/def (i.e. alternative or unlikely intentions) for the subsequent analyses.

#### Overt blocks

Participants performed the task well in both the baseline and the bias sessions (percentage mean correct responses: 98%, and S.D. = 2.5 and S.D. = 2.8). In the baseline session, RT analyses revealed a significant effect of the type of reputation, with participants being faster at inferring an action that was embedded within a tit-for-tat strategy than within an alternative strategy (e.g. always defect or always cooperate) (two-tailed *t*-tests, all p<.05). Crucially, this result confirmed the existence of an inherent preference towards TFT reputation. This effect was maintained after the bias assignment as revealed by faster RTs for the likely intention (i.e. the TFT intention) in the bias session (two-tailed t-tests, likely *vs.* unlikely intentions, all p<.001). However, RTs for likely intentions did not significantly decrease with time (blocks 1–3 *vs.* blocks 4–6: t = 0.35, p>.05; blocks 4–6 *vs.* blocks 7–9: t = −0.68, p>.05) (see figure 3, ‘Social Basic exp.’).

#### Covert blocks

**Figure 6:**
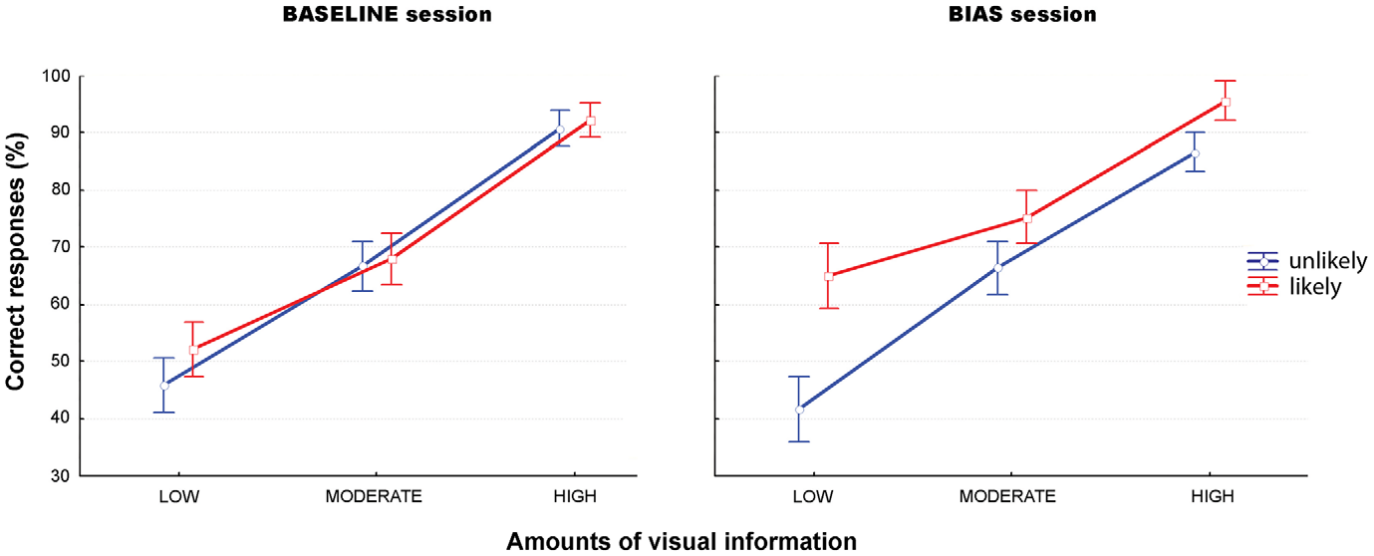
Social basic intention experiment (COVERT blocks). Mean percentage of correct responses (± SD) for likely (red) and unlikely (blue) intentions for each amount of visual information (low, moderate, high).

*Baseline session*. ANOVAs performed on both hits and RTs showed a significant main effect of the amount of visual information with decreased RTs (F(2,116) = 80.44, p<.001; 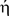 = .58) and a greater number of hits (F(2,116) = 209.02, p<.001, 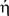 = .78) as the amount of visual information increased. In addition, the main effect of the type of reputation – previously observed in the overt blocks – was also significant in the covert blocks among RTs only (F (1, 58)  = 4.7, p<.05, 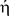 = .07). The second player's action was more rapidly inferred when it was embedded within a TFT intention than within an “always defecting” or an “always cooperating” intention. The type of reputation did not, however, interact with the amount of visual information (see table 3 and figure 6, ‘Baseline session”).

**Table 3:** Social basic intention experiment (COVERT blocks).

Experiment		Hits (%)			RTs (ms)		
Session	Intention	LOW	MODERATE	HIGH	LOW	MODERATE	HIGH
SOCIAL BASIC							
Baseline	Unlikely	45.8±13.4	66.6±11.7	90.8±9.3	1159±263	990±220	888±210
	Likely	52.2±12.5	68±12.3	92.2±7.5	1053±232	899±213	750±169
Bias	Unlikely	41.6±17.3	66.3±14.4	86.6±11.6	1217±297	1041±282	788±229
	Likely	64.9±13.7	75.2±10.6	95.5±6.4	846±220	748±216	607±158

Mean reaction times (*±* SD) for likely and unlikely intentions for each amount of visual information (low, moderate, high).

*Bias session*. There were significant main effects of the amount of visual information and of the bias on both the RTs (main effect of amount of visual information: F(2,116) = 114.49, p<.001, 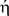 = .66; main effect of the bias, F(1,58) = 25.29, p<.001, 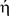 = .3) and the hits (main effect of amount of visual information: F(2,116) = 170.34, p<.001, 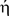 = .74; main effect of the bias, F(1,58) = 34.75, p<.001, 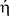 = .37), as well as a significant effect of the interaction between these two factors (RTs: F(2,116) = 9.23, p<.001, 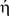 = .13; hits: F(2,116) = 8.28, p<.001, 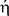 = .12). Participants' performance (slower RTs and higher hits) for actions congruent with a biased (i.e. likely) social intention improved as the amount of visual information decreased. Furthermore, the bias significantly affected participants' hits for all amounts of information (LSD Fisher: low, p<.001; moderate, p = .005; high, p =  *ns* for RTs; low, p<.001; moderate, p = .05; high, p = .05 for hits) (see table 3 and figure 6, ‘Bias session’).

#### Effect of the bias on the unlikely intention

When comparing performance for unlikely intentions between the baseline and the bias sessions, we found significant differences between these sessions for a high amount of visual information only, with participants' RTs for unlikely intentions significantly decreasing in this condition (two-tailed t-tests: t(30) = 2.26, p = 0.03).

#### Preliminary discussion

Two results make the present experiment diverge from the two previous ones. First, in the baseline session, where no bias was assigned, participants were faster at predicting an action associated with a tit-for-tat (TFT) intention. Interestingly, this early preference for TFT strategies differed from several aspects of the probabilistic bias that was imposed on participants in the second session. Not only did this preference not interact with the amount of visual information but its effect on performance remained constant over time. Second, the effect of the probabilistic bias was significant for any amount of visual information showing that prior expectations contributed to the inference even in conditions in which the visual information was highly reliable. By increasing the probability that the second player does whatever the opponent did in the previous round, we forced participants to perceive the second player as a ‘tit-for-tat’ player, rather than an altruist (always cooperate), an egoistic (always defect), or a ‘random’ player, hence generating progressively reinforced expectations that might prevail on relevant perceptual cues – as it is the case in conditions of high amount of visual information.

### Experiment 4: social superordinate intention

Statistical analyses were similar to those conducted in the first three experiments. Two-tailed t-tests revealed no significant differences among both participants' RTs and hits between the two likely combinations (coop/coop *vs*. def/def: two-tailed *t*-tests, all p>.2) and between the two unlikely combinations (coop/def *vs.* def/coop, two-tailed *t*-tests, all p>.15). Performances for def/def were therefore pooled with those of coop/coop (TFT or likely intentions) and performances for def/coop were pooled with those of coop/def (unlikely intentions).

#### Overt blocks

Participants performed the task well in both the baseline (percentage mean correct responses: 97.5%, S.D. = 2.8) and the bias sessions (percentage mean correct responses: 98.4%, S.D. = 2.6). In the baseline session, RT analyses revealed a significant effect of the type of reputation (two-tailed *t*-tests, all p<.05), with participants being faster at inferring an action that was embedded within a tit-for-tat strategy than within an alternative strategy (i.e. always defect or always coop). RTs for the likely intention also significantly decreased in the bias session (two-tailed t-tests, likely *vs.* unlikely intentions, all p<.001) and this decrease was found to increase over time up to block 6 (minimal RT = 369 ms), (blocks 1–3 *vs.* blocks 4–6: t = −2.11, p<.05; blocks 4–6 *vs.* blocks 7–9: t = −0.44, p>.05) (see figure 3, ‘Social superord. exp.”).

#### Covert blocks

**Figure 7:**
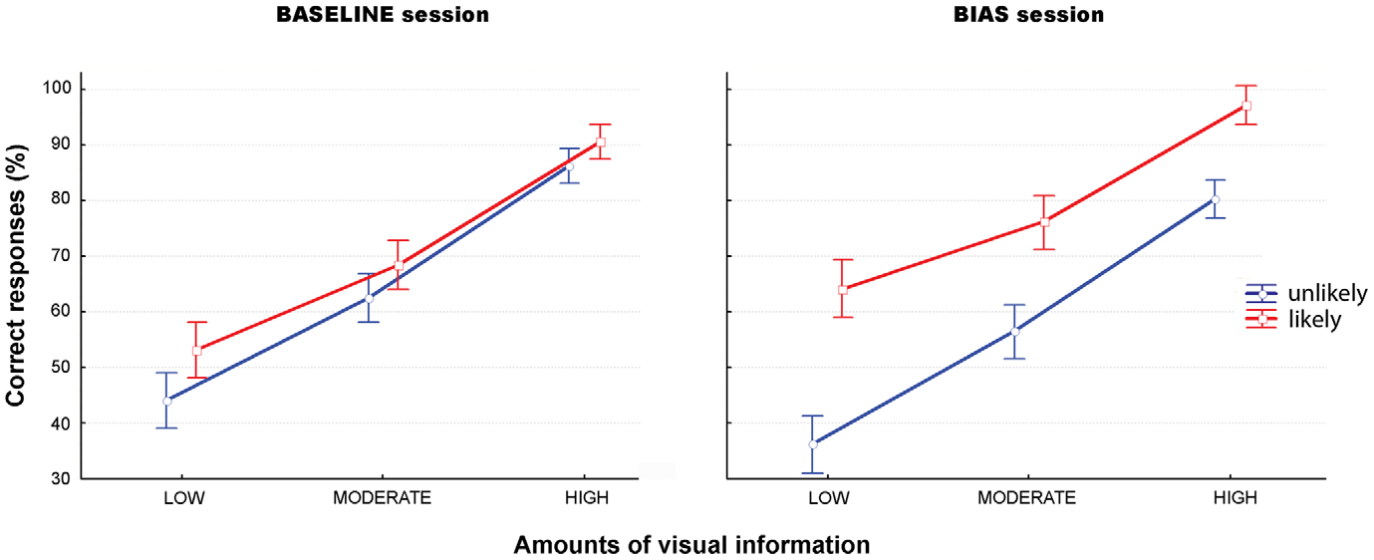
Social superordinate intention experiment (COVERT blocks). Mean percentage of correct responses (± SD) for likely (red) and unlikely (blue) intentions for each amount of visual information (low, moderate, high).

*Baseline session*. As the amount of visual information increased, decreased RTs (F(2,116) = 93.23, p<.001, 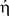 = .61) and increased hits (F(2,116) = 281.6, p<.001, 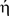 = .82) were observed for actions accomplishing the likely social intention. In addition, as in the overt blocks, actions were better and more rapidly inferred when they were embedded within a tit-for-tat strategy than within an alternative strategy (RTs: F(1,58,) = 4.16, p<.05, 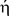 = .06, and hits: F(1,58) = 7.96, p<.01, 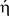 = .12). The effect of reputation did not, however, interact with the amount of visual information showing that the type of strategy affected participants' performance independently of the amount of visual information (see table 4 and figure 7, ‘Baseline session’).

**Table 4:** Social superordinate intention experiment (COVERT blocks).

Experiment		Hits (%)			RTs (ms)		
Session	Intention	LOW	MODERATE	HIGH	LOW	MODERATE	HIGH
SOCIAL SUPERORD.							
Baseline	Unlikely	44.1±14.5	62.4±11.5	86.1±9.1	1282±288	1049±237	877±198
	Likely	53±12.7	68.3±12.2	90.5±7.8	1102±277	957±217	816±175
Bias	Unlikely	36.1±15.2	56.3±14.6	80.2±11.8	1488±328	1197±271	961±230
	Likely	64.1±12.2	76.1±11.7	97.2±5.9	923±219	785±192	704±151

Mean reaction times (*±* SD) for likely and unlikely intentions for each amount of visual information (low, moderate, high).

*Bias session*. There were significant main effects of the amount of visual information and of the bias on both the RTs (main effect of amount of visual information: F(2,116) = 163.11, p<.001, 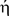 = .73; main effect of the bias F(1,58) = 52.03, p<.001, 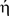 = .47) and the hits (main effect of amount of visual information: F(2,116) = 198.77, p<.001, 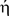 = .77; main effect of the bias F(1,58) = 92.16, p<.001, 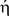 = .61), as well as a significant effect of the interaction between these two factors (RTs: F(2,116) = 27.74, p<.001, 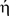 = .32, and hits: F(2,116) = 4.41, p = .01, 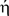 = .07). Participants' performance (slower RTs and higher percentage of hits) for likely social intentions improved to a large extent as the amount of visual information decreased, although the bias effect was observed for all amounts of visual information as revealed by the Post-hoc (LSD Fisher: low, p<.001; moderate, p<.001; high, p = .01 for RTs and low, p<.001; moderate, p<.001; high, p<.001 for hits) (see table 4 and figure 7, ‘Bias session’).

#### Effect of the bias on the unlikely intention

We found that reaction times for unlikely intentions significantly increased in the bias session, compared to the baseline session, for all amounts of visual information (two-tailed t-tests: all t(30)<−2.07, all p<0.05). The number of correct responses for unlikely intentions also significantly decreased in the bias session, for both low and high amounts of visual information (two-tailed t-tests: all t(30)>2.33, all p<0.02). This effect did not reach significance in the moderate amount of visual information condition (two-tailed t-test: t(30) = 1.8, p = 0.07).

#### Preliminary discussion

As for the social basic experiment, the bias effect was of major importance. First, in the baseline session, analyses of latencies and accuracy data revealed that, prior to the bias assignment, participants exhibited an early preference for social intentions congruent with a tit-for-tat reputation. Second, in the bias session, the bias significantly impacted on performances for all amounts of visual information revealing an increase in the difficulty with which the observer could disengage from his prior expectations, even in the case where these expectations interfered with the visual cues (e.g. in condition of high amount of visual information). Consistent with this interpretation is the observation that participants' hits for the unlikely intentions were found to significantly decrease in the bias session – as the selection of these unlikely intentions required inhibiting the activation of the likely intention representation.

### ‘Bias effect’: between-experiment comparisons

**Figure 8:**
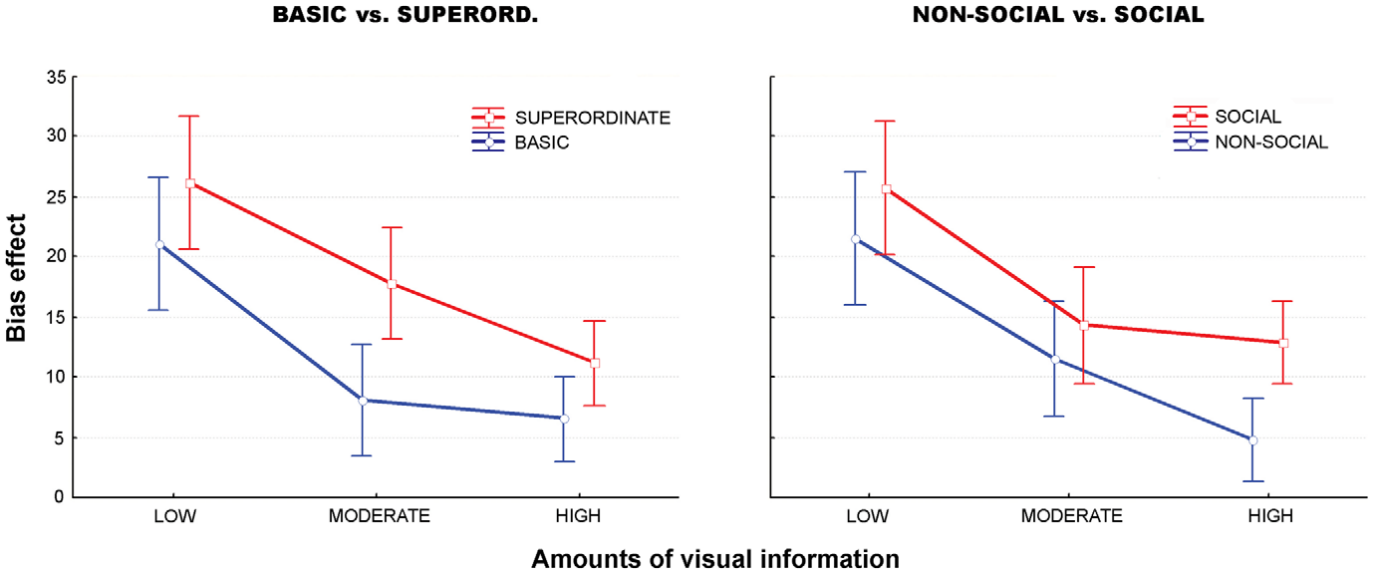
Mean score (± SD) of the bias effect expressed as a percentage of correct responses. **Left panel**: comparison between intentions with same target but different scopes (BASIC vs. SUPERORD.). **Right panel**: comparison between intentions with same scope but different targets (SOCIAL vs. NON-SOCIAL).

Contrasting the overall performance between experiments revealed no significant differences, showing that overall participants performed at comparable levels across the four experiments. Comparing the bias effect between experiments revealed significant effects of both the scope and the target of the intention. The bias effect was indeed significantly increased for the superordinate intention compared with the basic intention (F(1,116) = 8.36, p<.005, 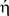 = .81) and for the social intention compared with the non-social intention (F(1,116) = 5.06, p = .02, 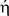 = .61). Furthermore, these differences were observed for different amounts of information according to the dimension itself. Indeed, along the scope dimension, the bias had a significantly greater effect when inferring a superordinate intention than a basic one in the conditions with a moderate amount of visual information (post-hoc Fisher test, p = .014). Along the target dimension, on the other hand, the only significant difference was observed for a high amount of visual information with a greater bias effect for inferring social intentions than non-social ones (post-hoc Fisher test, p = .0035) (see figure 8).

#### Preliminary discussion

Comparing the bias effect across the four experiments, two main results emerged. First, consistent with the previous results, we found that the bias differentially affected performance according to the scope and the target of the intention. The bias effect was indeed significantly more important in superordinate conditions than in basic ones and in social conditions than in non-social ones. Second, this effect varied according to the amount of visual information available to the participants. It was significantly more important for superordinate intentions than for basic intentions in condition of moderate amount of visual information and greater for social intentions compared to non-social intentions in condition of high amount of visual information.

## Discussion

The present study aimed at investigating how two distinct sources of information – perceptual (bottom-up) evidence and *prior* (top-down) expectations – interact to enable one to make an intentional inference. To do so, we manipulated the participants' prior expectations about the probability of the underlying intention while varying the amount of visual information in the action scene. Our second purpose was to determine whether the contribution of these two sources of information would vary depending on the scope (basic vs. superordinate intention) and the target of the intention (social vs. non-social intention) that had to be inferred. To test this second hypothesis, we therefore manipulated the type of intention underlying the observed action using four distinct tasks (basic non-social, superordinate non-social, basic social, and superordinate social tasks).

Two main results emerged. First, we observed that the intentional judgment indeed rests on an interplay between the participants' prior expectations (their probability of being true varied across the blocks) and the reliability of the sensory information available from the action scene. When this reliability decreased, the bias effect (i.e. the contribution of prior expectations) on performance increased, with participants responding more towards intentions they estimated as being the most likely cause of the observed behaviour. Second, this interaction was found to vary according to the *type* of intention, defined here by its scope (basic vs. superordinate) or its target (social vs. non-social). Indeed, directly comparing performance between intentions of different scopes but identical targets, and between intentions with the same scope but distinct targets, revealed an increase in the bias effect for both superordinate and social intentions. While this effect was only observed when the amount of visual information was low in the basic task, it was found to be significant for both low and moderate amounts of information in the superordinate task, and for any amount of visual information in the social conditions.

Taken together, these results indicate that the degree to which the participants' prior knowledge contributes is sensitive to the type of intention that is focused on. As the intention being considered becomes more abstract (from basic to superordinate, and from non-social to social intentions), the inference problem becomes less constrained (i.e. the number of intentions congruent with visuomotor inputs increases): in this condition, participants' prior expectations exerted an increasing influence on their responses, to the detriment of the sensory information available from the action scene.

### Interaction between perceptual and prior information

In the 4 experiments in the present study, the degree to which prior expectations contributed strongly depended on the reliability of the visual information conveyed by the video scenes. In low amount of visual information conditions, whatever the type of intention, participants tended to give priority to likely intentions at the expense of unlikely intentions; that is, they relied mostly on the intention they estimated to be the most likely cause of what was observed. This tendency towards favouring prior knowledge over perceptual information may further be accounted for by considering intentional inference as an *inverse problem* [1]–[3], [14]. Inverse problems characterise situations in which the same sensory input can have different causes. This type of problem is commonly encountered in ambiguous perceptual tasks – such as those using bi-stable or degraded stimuli – the resolution of which requires appealing to prior knowledge or making further assumptions about the nature of the observed phenomenon [43]. The significant contribution of prior expectations in conditions of high visual ambiguity precisely suggests that when sensory information was not sufficient to unambiguously infer one intention, participants compensated by massively appealing to their prior knowledge (i.e. about the space of the agent's possible intentions). This strategy resulted in preferentially selecting actions achieving the intention with the highest probability to occur.

Overall, this result reinforces the idea that in situations of sparse or incomplete data a successful inference depends on an adaptive integration between bottom-up information (from the observation of behaviour) and top-down prior knowledge about goals or intentions [13]. This integration is consistent with a mechanism complementing the available perceptual information when it does not sufficiently constrain the number of potential solutions, namely, the many competing intentions congruent with what is observed. In line with this assumption, some authors have suggested that inferring another person's intention necessarily requires sensorimotor information to be complemented with information about mental states and attitudes [23]. It has been demonstrated that prior expectations are already used frequently by children, even at a very early age. This tendency combines with a tendency to interpret actions as being directed towards a goal (‘teleological obsession’, [3]). When the visual information is not sufficient for interpreting the action as a goal directed one [44], or when the action is incomplete [45], children posit states of the world occasionally counterfactual to the perceptual evidence (such as the presence of occluded physical objects). The results of the present study are consistent with the existence of such a mechanism of data completion/correction operating through the default use of prior expectations. Crucially, however, we further show that reliance on this mechanism also depends on the type of the intention to be inferred, according to its scope (basic vs. superordinate) or its target (non-social vs. social).

### Basic vs. superordinate intentions

Both non-social basic and superordinate experiments required recognising one motor act, with the superordinate condition also requiring the final goal of the sequence (i.e. the shape being constructed) to be taken into account. Yet, across both experiments, prior expectations were found to differentially contribute to the participants' responses. In the basic non-social experiment, a bias response towards the likely intention was only observed in the condition where the amount of visual information was low. When participants were exposed to a moderate amount of visual information, these expectations no longer exerted an influence on performance, which then substantially depended upon the processing of the visual information alone. On the other hand, a heightened contribution of these expectations is observed in the superordinate experiments since they significantly influenced participants' performance in conditions of both low and moderate amounts of visual information.

The increase in response bias in the superordinate experiment cannot be explained by differences in complexity between both tasks since participants performed at comparable levels across basic and superordinate experiments showing that the differences between both experiments in terms of contribution of prior information are accounted for by the type of intention being considered. This result may be explained by differences in the relationship between these two types of intentions and action. While basic intention stands to action in a one-to-one relation (basic intentions like ‘transport’, ‘rotate’, or ‘lift’ an object are indeed directly accessible to the viewer from mere observation of the motor acts), superordinate intention stands to action in a many-to-one relation since the very same intention can be achieved by several distinct (commutative) sequences of actions. In the present study, this commutative property resulted in an ability of participants to infer the underlying intention solely on the basis of visual information arising from the first two actions. However, the present results also suggest that, despite the unpredictability of the sequence, participants still initiated a response, before observing the last action, by appealing massively to their prior expectations. Participants' dependence on priors in this condition could precisely account for the fact that *simulating* the motor acts composing the sequence (through motor mirroring, [46]–[47]) was of little help to infer the final superordinate intention. Those motor acts were indeed interchangeable within the sequence itself, and, as a consequence, they did predict neither the subsequent action nor the intention eventually achieved.

This early use of prior expectations might be accounted for by the existence of a system that pre-processes the current action chain depending on the sequences previously encountered. Observing the beginning of an action, or a sequence of actions, would automatically activate a representation of the likely intention that would be progressively suppressed or reinforced as the amount of visual information increases. Such a pre-processing would be particularly salient in superordinate conditions, where the beginning of the act chain proved to be of little importance for inferring the final intention it achieved. As such, it would explain why selecting an unlikely intention in bias sessions induced a significant cost on participants' performance. In these sessions, selecting an unlikely intention would indeed imply disengaging from the early activation of a likely intention. Finally, such pre-processing may account for why prior expectations are favoured over visual information in conditions of moderate perceptual uncertainty, as it would account for the role that priors continue to play when the amount of perceptual information increases. In superordinate conditions, the current sequence of actions would pre-activate the representation of the likely intention (i.e. the intention with the highest probability) to such an extent that a greater amount of visual information would be required to counteract it.

### Non-social vs. social intentions

Social experiments were characterized by participants' responses over-relying on prior expectations as revealed by responses massively shifting towards likely intentions (i.e. ‘tit-for-tat” intention: cooperation if previous cooperation, defection if previous defection) whatever the amount of visual information available from the action scene. This increased reliance on prior knowledge cannot be accounted for by differences in terms of complexity between non-social and social experiments, namely a greater memory load due to the requirement of tracking two successive intentions – the first and the second player's ones. Indeed, participants performed equally well, in terms of correct responses and reaction times, in both the social and non-social experiments. Additionally, the effect of facilitation associated with TFT strategy in the basic social experiment cannot be explained by a visual priming effect of the first player's action on the second player's one, which could have occurred when the latter performed the same action as the former. Indeed, TFT strategy was also found to be favoured in the superordinate social experiment; yet in this study TFT strategy did not necessarily imply that the action of the first player should be reproduced by the second player.

The dependence of the participants on their prior knowledge appears to reflect some expectations driven by the social context of the task. It is well-known that even basic movements, like the relative movements of geometrical figures, automatically induce participants to perceive the figures as socially interacting [48]–[50], and elicit strong expectations about the intentional causes of their movements (e.g. striking, kissing, etc.). Situations identified as involving social interactions are generally prone to trigger specific expectations concerning the way agents are likely to behave in such situations [12]. These expectations may be derived from perceiving the other as an interaction partner rather than a competitor in a joint-action task [51], and from knowledge of diverse origins, such as that provided by group stereotypes [52], social-specific naive theories [53], or an individual's reputation acquired from experience of reciprocal social interactions [10]. In the present experiment, increased dependence on prior knowledge for inferring social intentions, regardless of their scope, seems precisely to fall within this type of expectation. Indeed, during the whole task, increasing the frequency of the second player adopting a TFT strategy amounted to progressively assigning a specific reputation to that player. A bias in the response towards a “tit-for-tat” mode of reciprocation reveals that participants did integrate this reputational knowledge and made their response accordingly.

The pervasive effect of these specific expectations is also well illustrated in the baseline condition by the early preference of participants for the TFT reciprocation. Even before being biased in this direction, participants tended to infer more rapidly that the second player was more inclined to mirror the first player's strategy. This early preference was probabilistically reinforced in the bias session and, as a consequence, exerted a greater influence on the participants' performance since it persisted even when the reliability of the visual information was high. Indeed, while in the basic non-social experiment the very same motor act presented alone was inferred from a much lower amount of visual information, in the social experiment a bias response towards the likely social intention was still observed for a higher amount of visual information. This shows that the influence of these expectations in the bias session was such that the participants had difficulty disengaging from their *a priori* expectations, resulting in predicting a play congruent with prior expectations but counterfactual to perceptual evidence. Similarly, these difficulties could account for the cost in performance associated with the selection of intentions that did not meet these expectations. In the bias session, participants were indeed significantly less accurate and were slower to select an unlikely intention (i.e. always defect, always cooperate) when this selection required concomitantly inhibiting the competing tit-for-tat intention.

### Simulation vs. reasoning accounts of action understanding

The two main results of the present study (interaction between prior expectations and perceptual information and the modulation of this interaction as a function of the type of intention) may help reconcile the two major accounts of action understanding developed over the last decade. On the simulation account, we understand our conspecifics' intention by literally simulating their action via the activation of our own motor planning system. The result of this process of internal replication is the selection, in the observer's own repertoire, of the intention that would have caused the very same action. This type of explanation stresses the role of sensory information, derived from the kinematics of the movement, in action understanding [35]– irrespective of whether the action is complete (the goal achieved is fully visible) or only partially performed (the goal is hidden but can be predicted from the unfolding action) [see 46]. In contrast, the “theory theory” account postulates that action understanding is based on specialized inferential processes and mostly emphasizes the contribution of the context-related prior knowledge. This knowledge can either be derived from our intuitive theories of human behaviour, or from the subject's past experiences and rules she has drawn from them [8], [54], [55].

A wealth of empirical data and theoretical works nowadays converges on the idea that these two major classes of mechanisms play a complementary role in intention inference [10], [22], [23], [56]. The results of the present study comfort these observations. By suggesting that intentional judgment relies on a relative balance of bottom-up sensory and top-down prior information, they plead in favour of a *hybrid* model of action understanding. In such a model, the observer would mobilize either low-level simulation or high-order inferential mechanisms depending on whether the current sensory evidence is, or is not, reliable enough to elicit simulation from observation.

Recently, Kilner and colleagues proposed a theoretical framework that attempts to further account for how these two classes of mechanisms may interact to enable one's understanding of other people's intentions [1]. This framework relies on the hierarchical architecture of action representations ranging from the intention level to the kinematics level (see also [57]). In this architecture, the selection of one type of action representation would result from the resolution of the inverse problem at each level of the hierarchy. Basically, each level uses a model to generate a prediction of the representations in the level below. This prediction is then compared with the representation at the subordinate level and prediction errors arising from that comparison are returned to the higher level to adjust its representation. This adjustment is generalised to the different levels of the hierarchy (intention, motor command and kinematics). The most likely cause of the observed action is then inferred by minimising the prediction error at all the levels of this hierarchy [1], [2]. Given visual kinematics, goal expectations are first generated, from these goal representations motor commands are then predicted and given these motor commands, kinematics are in turn predicted. In this framework, top-down influences are therefore dynamically generated since the estimates produced at the higher levels become prior expectations for the lower levels.

Our results can be consistently interpreted in the light of the Kilner's hierarchical model. A basic intention can be directly predicted from the observation of the current motor act, provided the related visual information is sufficient to enable comparison with expected kinematics at higher levels. In this case, participants' performance is strongly dependent on minimising the prediction error that arises from this comparison. However, this comparison also closely depends on the reliability of the current movement kinematics; when the amount of visual information is too low, this comparison cannot be made, and, as a result, subordinate levels cannot adjust their representation to higher estimates of the hierarchy. We observed that, when this comparison could not be carried out, participants consistently appealed to their prior knowledge. In a hierarchical model of action representations, such an over-reliance on priors could be made possible by the existence of a short circuitry of recursive loops between subordinate and higher levels of the cortical hierarchy. These recursive loops would be mobilized when data is sparse to shortcut the automatic comparison process between observed and expected kinematics movement. Importantly, the engagement of this mechanism proved to be dependent on the amount of visual information available from the action scene, but independent from the scope and target of the intention, since it was observed to operate at the lowest levels of visual information in each of the four experimental conditions.

Noteworthily, the engagement of these recursive loops is also sensitive to variations in the relationship between the observed action and its goal. Superordinate conditions indeed involved a greater recourse to participants' prior expectations even when the visual information significantly increased to a moderate (non-social) or even a high level (social). This greater dependence on prior expectations can be explained by the fact that, in superordinate conditions, many competing intentions are congruent with the visual information conveyed by the current motor act. Thus, whereas minimising the prediction error between expected and current kinematics may be sufficient to predict the agent's single act (e.g. to rotate), it may not be to infer unambiguously which of the multiple superordinate intentions (e.g. final shapes) it contributes to accomplish. As a consequence, we found the weight of the decision to be mostly carried by participants' prior expectations, suggesting, in this situation of accrued perceptual uncertainty, an early shortcut of the comparison process between levels of the action representation hierarchy. Crucially, this shortcut was independent of the amount of information, since it occurred even when the visual information was high enough for the participant to be normally confident about what she is seeing. This observation suggests that recursive loops of this kind could be mostly recruited in contexts where relying on one's prior expectations is a better guarantee for accurate inference, even if such expectations can occasionally go against perceptual evidence.

### Conclusion

Our results shed light on how sensory information, derived from the kinematics of the observed action, interacts with prior expectations to enable one's understanding of other people's intentions. We first showed that the contribution of participants' prior knowledge was sensitive to the availability of the sensory information from the action scene. A greater contribution of this knowledge was observed in conditions of sparse visual information, suggesting the engagement of a mechanism of data completion operating through the default use of prior expectations.

Second, we found that the priors' contribution also depended on the type of intention that was inferred. An increased reliance on priors was indeed observed in conditions where the agent's intention could not be predicted by the sole visible, current motor act, but further required estimating the superordinate goal this act contributed to achieve. In this case, participants' expectations – being progressively acquired from observation – were found to most frequently supersede the visual information conveyed by the current motor kinematics. Thus, the more participants responded towards the biased (e.g. expected) intentions, the more the visual information tended to play a confirmatory, rather than a predictive role. Such a shift in the contribution of visual evidence is likely to account for why participants, in this condition, mostly over-relied on their priors to make their decision, even though it ran counter to the perceptual evidence.

Crucially, an over-reliance on priors was also massively observed in social conditions. We suggested that the early influence of social-specific expectations (e.g. expectations on how agents are the most likely to behave in a context of reciprocal interaction) may account for this important shift in the response toward participants' priors. Contexts of social interaction are indeed prone to elicit modular, high-level expectations, which may contribute to giving priority to some intentional causes (e.g. cooperation if previous cooperation, defection if previous defection) at the expense of other competing causes. These *a priori* expectations, being acquired from experience (probabilistic bias) or derived from domain-specific knowledge (TFT reciprocation), were found to favour some action representations so that less sensory evidence was needed for the participants to be confident about their decision, *i.e.* about which kind of intention was most likely the cause of the observed action.

## Supporting Information

Text S1 Pre-tests: intra- and inter-sequence comparisons; selection of low, moderate and high amounts of information.
(DOC)

Figure S1 **Distribution of participant's reaction times (blue dots) across the 12 movie segments**. Reaction times for the different actions were pooled across subjects. Red squares: mean reaction times across participants for each of the 12 duration ranges.
(TIF)

Figure S2 **Basic experiment: psychometric curve fit to the cumulative distribution of participant's correct responses (red dots).** Responses for the different actions were pooled across. The blue dot refers to the inflexion point of the sigmoid curve. In each experiment, the inflexion point occurs at the following duration: **A**. Basic: 1576 ms. **B**. Superord.: 1558 ms. **C**. Social basic: 1546 ms. **D**. Social superord. 1550 ms.
(TIF)
