# Supplementary material for: What Are They Up To? The Role of Sensory Evidence and Prior Knowledge in Action Understanding
Source: PLoS One. 2011 Feb 18;6(2):e17133. doi: 10.1371/journal.pone.0017133 (PMC3041795; doi:10.1371/journal.pone.0017133)
Supplement: Text S1 — Pre-tests: intra- and inter-sequence comparisons; selection of low, moderate and high amounts of information. (DOC) [file pone.0017133.s001.doc]

**Supplemental Material**

For each experiment, a pre-test was conducted in order to i) control for the temporal homogeneity of the action sequences, and ii) to select three temporal windows relevant for the subsequent experiments (i.e. low, moderate, high amounts). All movies were filmed using a digital camera (Sony®- HDR-SR7) and were acquired and tailored using the software Adobe Premiere®.

**1. Intra- and inter-sequence comparisons**

Prior to build each experiment, a pre-test was conducted to control the temporal dynamics of each action sequence. Specifically, we controlled that, for any point within a given sequence, a particular type (‘transport’ vs. ‘lift’ vs. ‘rotate’) or model (transport[1] vs. transport[2], lift[1] vs. lift[2], etc.) of action was not more or less discriminable than another one. To do so, each action scene in each of the 4 experiments was first cut at {960, 1160, 1320, 1400, 1480, 1560, 1640, 1720, 1800, 1880, 1960, 2080} ms after the onset of the movement. The resulting movies (12 clips per model of action) were randomly presented to a group of 12 participants in four sessions. Each session consisted in a basic action recognition task. In the two first sessions, movies from the basic and superordinate experiments were presented with the instruction of recognizing the performed actions by pressing, as quickly and as accurately as possible, one of the corresponding keyboard buttons. In the two last sessions, movies from the social basic and social superordinate experiments were shown with the same instruction. The order of sessions was counterbalanced across participants. For the (non-social) superordinate and social (basic and superordinate) movies, the only last action was shown.

Two groups of statistical analyses were conducted on both hits and RTs.

First, we compared participants’ hits for each type of action *within* each session (‘lift’ vs. ‘rotate’ vs. ‘transport’ actions within non-social basic and superordinate experiments; ‘transport’ vs. ‘rotate’ actions within social basic and superordinate experiments), then we compared hits for actions of the same type *between* sessions (e.g. lift[basic] vs. lift[superord.]).

Two-tailed t-tests revealed no significant differences between participants’ correct responses for the different types of action within each session (comparing ‘transport’ vs. ‘lift’ vs. ‘rotate’ actions in non-social basic and superordinate conditions, all *p*>0.22; comparing ‘transport’ vs. ‘rotate’ actions in social basic and superordinate conditions, all *p*>0.4), as well as no significant differences for actions of the same type between sessions (non-social conditions: lift[b] vs. lift[s], rotate[b] vs. rotate[s], transport[b] vs. transport[s]: all *p*>.19; social conditions: transport[b] vs. transport[s], rotate [b] vs. rotate[s]: all *p*>.25).

Second, we were compared the individual distributions of hits and reaction times (RTs) across the different movie segments to ensure that participants’ performance was equally sensitive to variations in the amounts of visual information (see **Supporting Figure S1**). To do so, we computed within each experiment a one-way Kruskall-Wallis test with participants’ hits and RTs as dependent variables and the amount of information as a 12-level factor.

As shown in Figure S1, the participants’ detection curve showed the usual sigmoidal shape, performance rapidly increasing within a 1400-1700 ms interval of movie durations up to the maximal value (100%). In all experiments, Kruskall-Wallis tests revealed no significant differences between individual distributions of performance, with, between participants, identically decreasing RTs (basic: H(11,144)=9.34, *p*=.59; superordinate: H(11,144)=7.64, *p*=.74; social basic: H(11,144)=6.34, *p*=.84; social superord.: H(11,144)=5.1, *p*=.89) and increasing rate of correct responses (basic: H(11,144)=2.88, *p*=.9; superordinate: H(11,144)=2.61, *p*=.9; social basic: H(11,144)=1.98, *p=*.9; social superord.: H(11,144)=1.02, *p*=.9) as the available amount of visual information increased.

**2. Selection of low, moderate and high amounts of information**

For the needs of the subsequent experiments (social and non-social basic and superordinate exp.), 3 different amounts of visual information (corresponding to three different movie segments) were selected among the 12 controlled ones. To do so, a Weibull psychometric function was fit with the restricted least squares method to the data (we used a Weibull function because such function generally provides a good model for contrast discrimination and detection data ; see [58,59]). We then computed the amount of visual information corresponding to the inflexion point of the sigmoid curve within each experiment (shown as a blue dot on the resulting regression curves, see **Supporting Figure S2**). The duration corresponding to the inflexion point (1560ms – moderate) and the two nearest durations (1480 –, low and 1640 ms – high) were selected for the covert blocks. This choice ensured equal timing steps as well as similar performance differences between the three levels. The 1880 ms duration (≈100% hit rate) was chosen for the ‘overt’ trials.

The Weibull function is described by:

$$F(x;\alpha;\beta;\gamma)=1-\gamma\exp\left[-\left(\frac{x}{\alpha}\right)^{\beta}\right],$$

$$0\leq x<\infty$$
