# Supplementary figures and images for: What Are They Up To? The Role of Sensory Evidence and Prior Knowledge in Action Understanding
Source: PLoS One. 2011 Feb 18;6(2):e17133. doi: 10.1371/journal.pone.0017133 (PMC3041795; doi:10.1371/journal.pone.0017133)

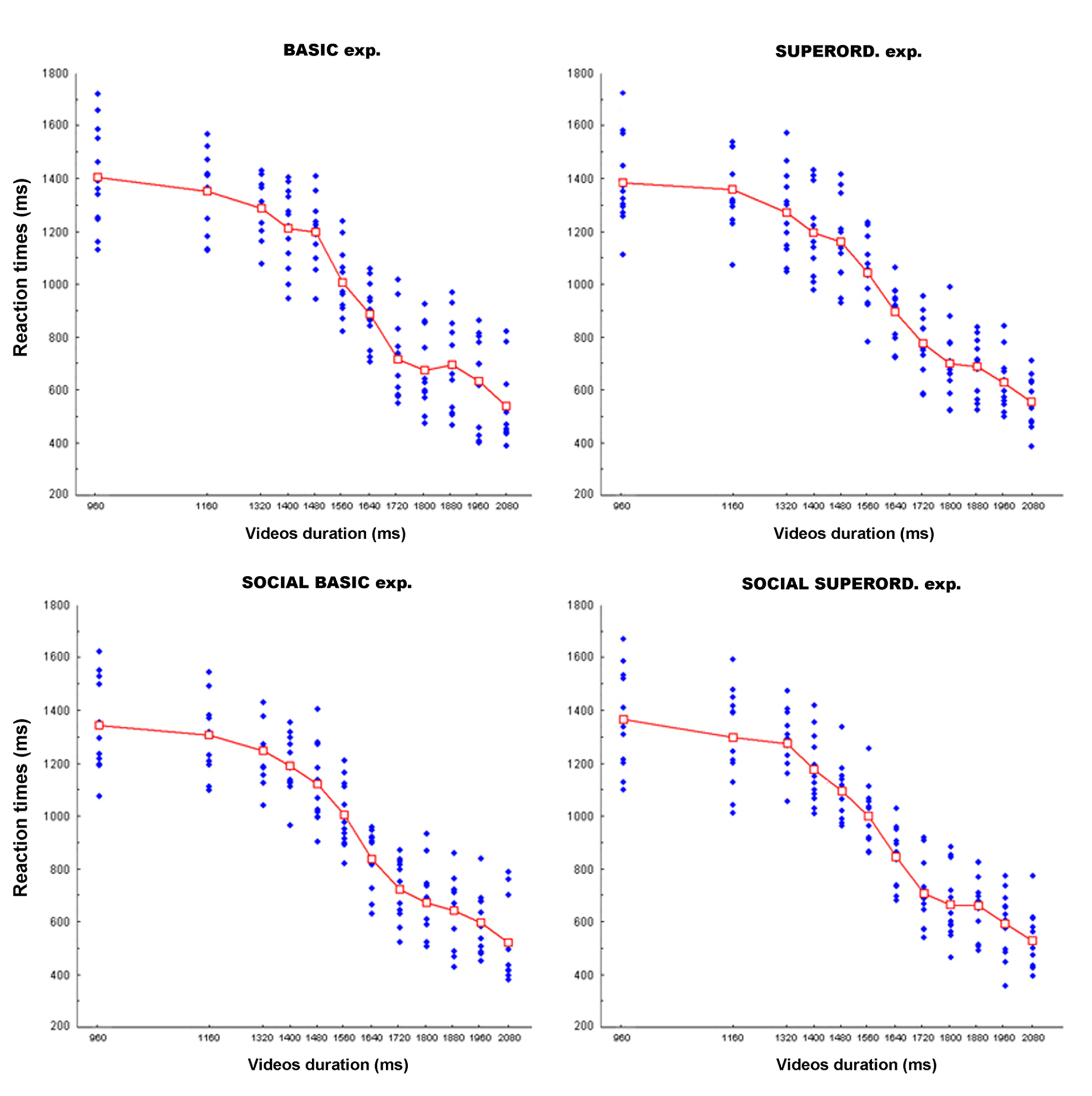

Supplement: Figure S1 — Distribution of participant's reaction times (blue dots) across the 12 movie segments. Reaction times for the different actions were pooled across subjects. Red squares: mean reaction times across participants for each of the 12 duration ranges. (TIF) [file pone.0017133.s002.tif]

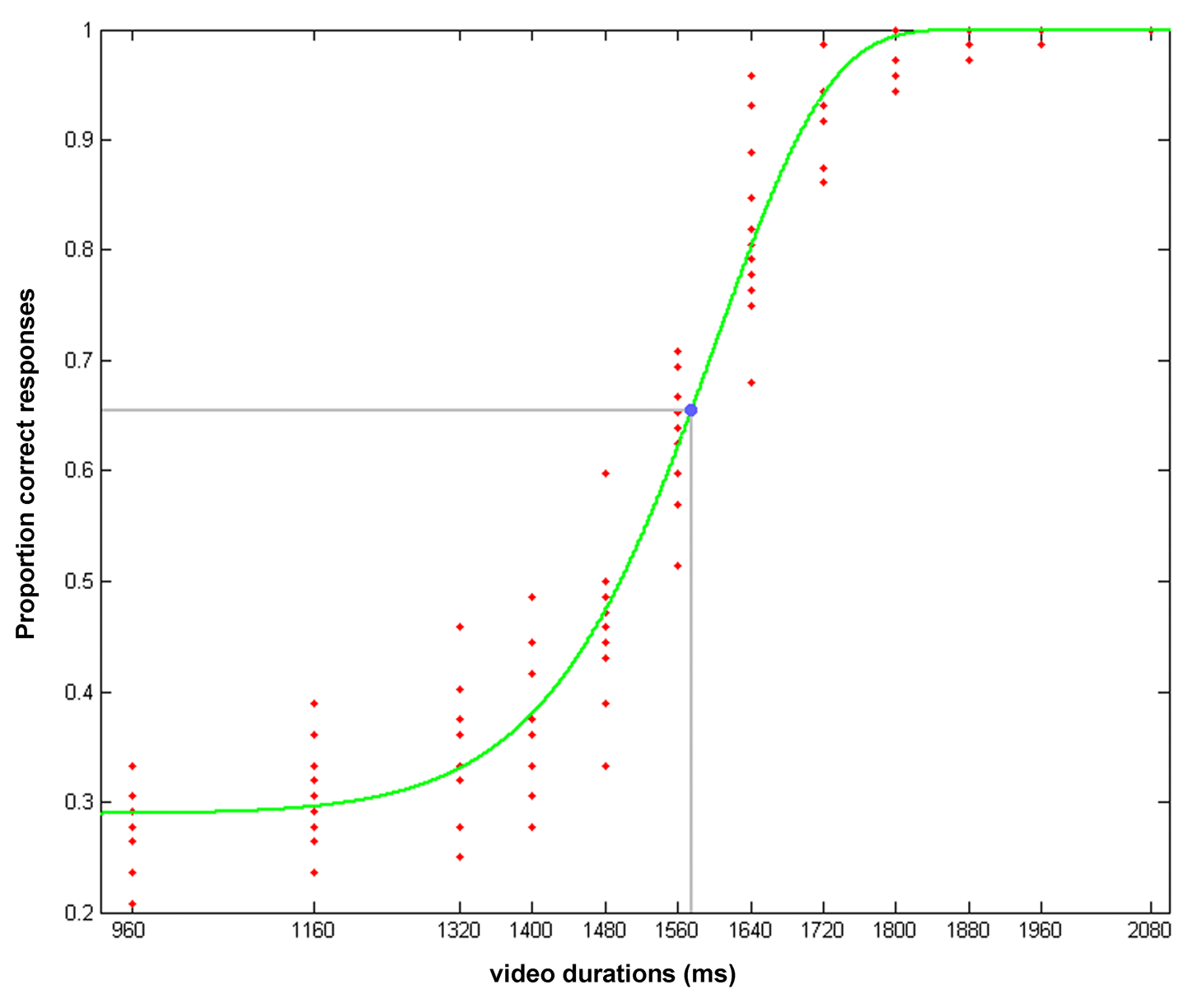

Supplement: Figure S2 — Basic experiment: psychometric curve fit to the cumulative distribution of participant's correct responses (red dots). Responses for the different actions were pooled across. The blue dot refers to the inflexion point of the sigmoid curve. In each experiment, the inflexion point occurs at the following duration: A. Basic: 1576 ms. B. Superord.: 1558 ms. C. Social basic: 1546 ms. D. Social superord. 1550 ms. (TIF) [file pone.0017133.s003.tif]
